# The influence of stimulus format on drawing—a functional imaging study of decision making in portrait drawing

**DOI:** 10.1016/j.neuroimage.2014.08.015

**Published:** 2014-11-15

**Authors:** R.C. Miall, Se-Ho Nam, J. Tchalenko

**Affiliations:** a School of Psychology, University of Birmingham, Birmingham, B15 2TT, UK; b 47A Chevening Road, London SE19 3TD, UK

**Keywords:** Drawing, Visual processing, Decision-making, fMRI, Top-down modulation, Face processing

## Abstract

To copy a natural visual image as a line drawing, visual identification and extraction of features in the image must be guided by top-down decisions, and is usually influenced by prior knowledge. In parallel with other behavioral studies testing the relationship between eye and hand movements when drawing, we report here a functional brain imaging study in which we compared drawing of faces and abstract objects: the former can be strongly guided by prior knowledge, the latter less so. To manipulate the difficulty in extracting features to be drawn, each original image was presented in four formats including high contrast line drawings and silhouettes, and as high and low contrast photographic images. We confirmed the detailed eye–hand interaction measures reported in our other behavioral studies by using in-scanner eye-tracking and recording of pen movements with a touch screen. We also show that the brain activation pattern reflects the changes in presentation formats. In particular, by identifying the ventral and lateral occipital areas that were more highly activated during drawing of faces than abstract objects, we found a systematic increase in differential activation for the face-drawing condition, as the presentation format made the decisions more challenging. This study therefore supports theoretical models of how prior knowledge may influence perception in untrained participants, and lead to experience-driven perceptual modulation by trained artists.

## Highlights

• Drawing natural images requires feature identification and extraction.
• Top-down decisions and prior knowledge e.g. of faces guide this process.
• We used fMRI with concurrent eye tracking and measured drawing actions.
• Ventro-lateral occipital areas were more active drawing faces than abstract objects.
• There was also differential activity with more difficult stimulus formats.

## Graphical abstract

**Figure:**
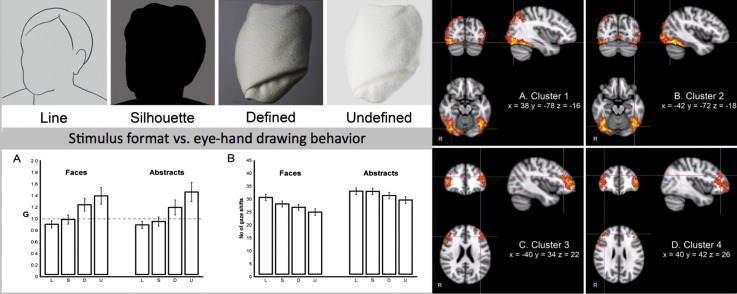

## Introduction

When drawing pictures, whether from life or from memory, or when copying photographs or paintings, there are complex decisions to be made in order to allow the rendering of an original image into a discrete series of pencil or pen strokes on paper. Only if copying or tracing an existing line drawing are these decisions avoided. But when for example, making a line drawing of a face, or a photograph of a face, features of the face have graded light or color intensities—perhaps the graded border between the cheek and the nose—that need to be caught as singular lines. As Perdreau and Cavanagh (2013) have recently discussed, there is a long chain of neural transformations between the initial processing of the image that falls onto the retina, the perception of objects in the scene and spatial relationships between them, decisions about sub-features and boundaries within the objects, the selection of the line to be drawn, and ultimately, the sensory–motor control of the drawn line as the hand moves on the paper. It is the question of the decisions about features and boundaries and the selection of the line to be drawn that we focus on in this paper.

We have previously tested the eye and hand movements as naïve and expert artists draw portraits (Gowen and Miall, 2007, Miall and Tchalenko, 2001, Miall et al., 2009, Tchalenko and Miall, 2009). We have argued that in the interval between observation and selection of a feature of an image to be drawn, the chosen line is more likely stored as a motor plan than in a visual short-term memory (Miall et al., 2009). However, those studies did not allow us to investigate the processes involved in the selection of the drawn feature.

There is indeed a long but still active debate about the extent to which artists are able to isolate their perceptual judgments from both the perceptual distortions normally introduced into each sensory processing stream, and from existing knowledge about objects (e.g. Fry, 1981, Ostrofsky et al., 2012, Perdreau and Cavanagh, 2011, Ruskin, 1971). Thus our visual perception reduces the impact of perspective, illumination-induced color shifts, etc, and leads to biases in our judgments. Further, it is often difficult to avoid bringing prior knowledge about the observed objects to bear: naïve portraits typically distort the drawn face, over-emphasizing its canonical features—such as the two eyes being equidistant from the nose (Gombrich, 1960). Thus there is a strong interaction between cognitive and experience-driven prior knowledge and stimulus-driven perception. Seeley and Kozbelt (2008), for example, have argued that trained artists develop spatial schemata, and argue that premotor areas in the brain are responsible for the advantage in deploying these schemata as motor plans when drawing, thus overcoming the biases in visual perception. They propose “artists’ technical proficiency in a medium confers an advantage in visual analysis, which consists of the ability to focus attention on sets of stimulus features sufficient for adequate depiction”. Their model builds on previous theories (Kosslyn, 1996, Schyns, 1998), linking prefrontal attention areas with lower level visual processing areas—the perceptual features are extracted based on an iterative, hypothesis-testing loop between these frontal and temporal–occipital regions.

With this background, we aimed to study the neural underpinning of the act of drawing from photographic images, controlling both the level of stimulus ambiguity and the level of top down knowledge of the images. We contrasted drawing a prescribed feature of a face with a similar feature in an abstract object. We also controlled the ambiguity of this feature by presenting each image in four formats, from a line drawing to be copied, a silhouette, a low contrast and a high contrast photograph. We hypothesized that the decisions about how to draw each stimulus would be affected by the stimulus type (faces versus abstract), as the former would allow prior knowledge of faces to influence both the low level perceptual processes, but also influence the judgments about the ambiguous features. We therefore focus on the contrast faces vs. abstract, but briefly mention the reverse contrast. We also hypothesized an effect of stimulus format (line, silhouette, define and undefined), with the a priori expectation that the order of difficulty in making judgments about the lines would increase across that sequence of formats (Tchalenko et al., 2014). We further hypothesized that there would be an interaction between these two factors such that decision about drawing undefined faces might differentially engage cognitive processes compared to easy line drawing conditions of abstract objects. Finally, based on the model of Seeley and Kozbelt (2008), and on the underlying theories (Kosslyn, 1996, Schyns, 1998), we predict that prefrontal/premotor areas would have increased activation in the faces trials, based on the greater top-down knowledge of that category, while we predict that extrastriate areas would show a modulation in activity driven by this categorical knowledge and the increasing perceptual demand across the four presentation formats.

## Methods

### Participants

Fourteen participants were included in this study (mean age 30, range 24–41; all right-handed; 8 females). One additional participant was tested but excluded from the analysis as a result of a technical failure during the experiment. All participants gave written informed consent according to instructions and procedures approved by University of Birmingham Ethics Committee. The participants were not artists, art students, or recruited according to their drawing ability, and did not report any unusual history of drawing. All had normal vision, or vision corrected to normal with contact lenses.

### Material & apparatus

**Fig. 1:**
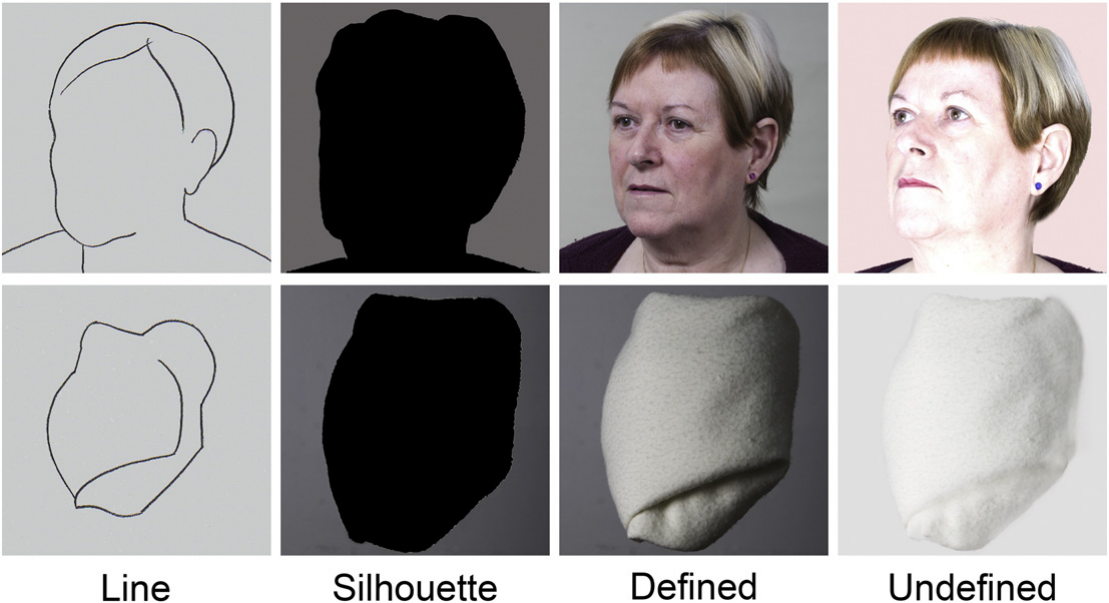
Examples of one set of faces and one set of abstracts (folded towels) used in the study. The horizontally or vertically flipped versions of these stimuli are not shown.

The task involved drawing the outline contour of images of faces or abstract objects (folded towels; Fig. 1). There were eight sets of faces, from eight individuals, and eight sets of abstract objects (different folding of the towel). Each set included four different formats, referred to as line, silhouette, defined (high contrast), undefined (low contrast).

The undefined and defined stimuli were derived from high resolution color photographs, which were individually manipulated using digital software to reduce or increase the contrast from the original, to ensure a poorly or well defined contrast of the face outline against the plain background. The line and silhouette stimuli were also individually produced by hand tracing the outline of photographs, filling with black for the silhouette stimuli (Fig. 1). Note that the corresponding photographs are not identical, but are recognizably the same face (or towel), from the same viewpoint.

To double the number of available stimuli without repetition, we presented each stimulus twice, once in the training session and once in the main experiment, in its original orientation or in a “flipped” version. To create the flipped version, the original faces were flipped horizontally, and the original abstracts were flipped vertically, to give a grand total of 128 face and abstract stimuli. There was no strong vertical orientation for the folded towel “abstract” images. Because of the fully balanced design of the experiment, in which every stimulus was used, these small variations in stimulus are expected to have no effect on the participants' decisions about the drawing task, but will maintain higher levels of attention and interest.

**Fig. 2:**
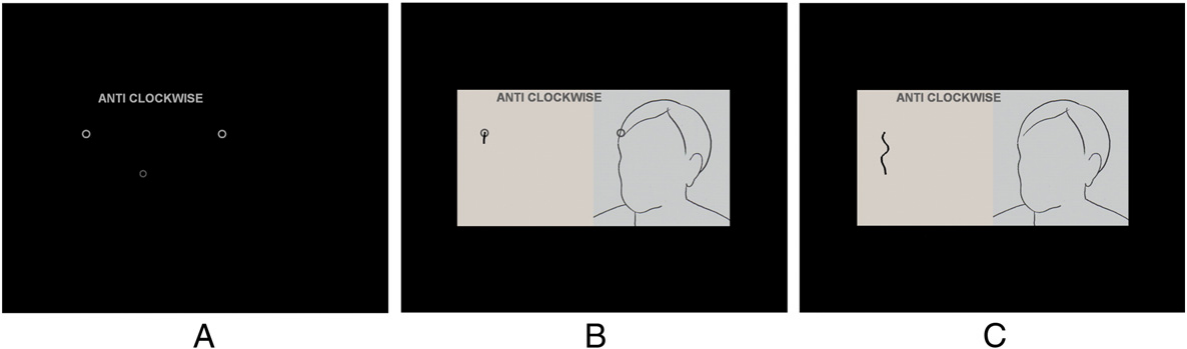
Experiment procedure. A: Participants were presented with two static circles, a flashing circle and a short message. The message indicated a drawing direction (ANTI-CLOCKWISE or CLOCKWISE) or if it was a ‘Rest’ trial. They were instructed to move the flashing circle, which represented the location of the drawing stylus, into the static circle on the left side to begin drawing. The static circle on the right showed a location of the image where they were to start drawing. B: After 3 s, an image was presented, and participants began drawing. C: The static circles disappeared after 3 s, and participants continued drawing for further 19 s. In a ‘Rest’ trial, only a short message (‘REST’) was presented for 3 s, and participants were instructed to look towards a fixation cross in the middle of the screen for 21 s.

Participants drew images in the MR scanner on a touch-sensitive panel using a stylus. The panel was constructed from a wooden frame (295 × 230 mm) housing an 8 wire resistive touch screen (255.0 × 190.0 mm, AMT/PN 9534). The participants looked at a visual display on a rear-projection screen positioned behind their head and viewed through a rear-view mirror with a viewing distance of proximately 600 mm. The visual display included the stimulus image frame (right side) and the drawing frame (left side) presented against a black background. The display subtended a horizontal and vertical visual angle of approximately 32 × 16 degrees (Fig. 2). Participants could see their drawings appear in real time as a black line in a light pink drawing frame, although they were not able to view their hand or the drawing panel. They held the drawing panel with their left hand supported by a pillow across their lap, and their knees were supported by a knee supporter.

The Long Range EyeLink 1000 eye tracking system combined with two separate infrared illuminators was used to record gaze positions of both eyes. The sampling frequency of the eye tracker was 250 Hz with an average accuracy of 0.5° of visual angle. A centroid model was adopted to fit the pupil image and determine the pupil position. A built-in nine-point calibration procedure was used, with randomly ordered presentation of the points. The calibration procedure was repeated until good calibration was indicated by the system. Calibration was also repeated before each 18 minute scanning run. A drift check and correction were carried out after calibration to maintain accuracy of the calibration parameters, and the gaze position accuracy was validated during fixations onto a central fixation cross displayed between trials.

The experiment was written in Matlab using the Psychophysics and Eyelink Toolbox extensions (Brainard, 1997, Cornelissen et al., 2002, Pelli, 1997; see http://psychtoolbox.org).

### Design/Procedure

Participants took part in both a training session and an experimental session. Before the main experiment, the training session was carried out while participants were lying within a mock scanner of same bore size to the magnetic resonance (MR) scanner, and with an identical head coil, mirror and projection screen. They were given verbal instructions in the mock scanner while they were performing the task, and if necessary given verbal corrections or reminders. Before entering the real scanner they were reminded about the instructions.

The experiment used a block design (or “slow” event-related design with single-trial blocks) with eight conditions, which comprised a 2 × 4 design (faces vs. abstract types, and line, silhouette, defined, undefined formats). A ‘rest’ condition was also included in the design as a baseline. Each scanning session composed of two runs, and each run contained 32 trials. In total 128 images (8 sets × 2 types [faces and abstracts] × 4 formats [line, silhouette, defined, undefined] × 2 versions [normal and flipped]) were pseudorandomized across the 128 trials of the practice and scanning sessions.

The order of normal and flipped presentation of each set were randomized and allocated evenly for both training and experimental sessions. Each session then included four different formats of each set, of which the half was a normal version and the other half was a flipped version. Each run included one format of a normal version and another format of a flipped version; if participants saw a normal version of any format in the training session, they would only see the flipped version of the stimulus in the experimental session and vice versa. Consequently, each run included two different presentations (one normal and one flipped) of each set of stimuli types, and each session included four different formats (half normal and half flipped) of each set of stimuli types. Thus, participants saw each stimulus only once during the whole experiment.

Each run started and ended with a ‘rest’ trial in which participants were requested to keep their eyes open and relaxed, looking towards a fixation cross in the middle of the screen, with no attempt to suppress blinks. In each run, a ‘rest’ trial was followed by a set of four trials, in which line, silhouette, defined and undefined conditions were randomized, and two of which were faces and two abstracts. Each run contained 41 trials (32 drawing trials and 9 rest trials), and each trial lasted 24 s. The run duration was therefore 16.4 minutes in total.

At the start of each drawing trial, participants were presented with two static circles, a smaller flashing circle and a short written message (‘ANTICLOCKWISE’, ‘CLOCKWISE’, or ‘REST’). The message indicated the required direction of drawing or if it was a rest trial and remained onscreen throughout the trial. The flashing circle represented the location of the drawing stylus. The static circle on the right pane showed the position on the original where they were to start the drawing from, while the corresponding circle on the left side was the start zone into which they were instructed to move the cursor to begin drawing. After 3 seconds of this initial display, a stimulus image was presented, and participants began drawing from the start zone. After a further 3 seconds, the static circles disappeared and the drawing was carried on for further 18 seconds (Fig. 2).

**Fig. 3:**
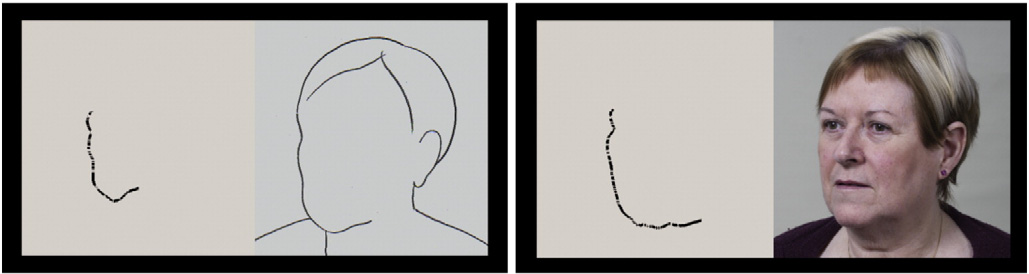
Two typical examples of drawing the border of the left cheek of the face shown in Fig. 2C, in line and defined formats.

For all formats of drawing trials, participants were encouraged to draw details of the contour of the image (including the cheek, chin and jaw for the face trials) as accurately as possible at a comfortable pace, drawing throughout the trial, and they were informed that finishing the drawing was not of importance. For defined trials, they were instructed to draw the well-defined contour against the background. For undefined trials, they were encouraged to use their judgment to draw the indistinct contour and were instructed not to draw any clearly defined parts of the image. Participants drew anti-clockwise in normal trials and clockwise in flipped trials, so that in all face trials they started to draw from near the hairline, down the cheek and towards the chin (Fig. 3).

### Behavioral data analysis

Drawing stylus positional data were collected at a sampling rate of 60 Hz and interpolated to match the sampling rate of eye movement data (250 Hz); through the calibration procedure both eye and stylus positions were defined relative to the screen coordinates in mm.

During active drawing, the participants' gaze would alternate between the right hand original (stimulus image) panel and the left drawing panel, and they typically made a few fixations in each panel before switching back to the other side. The number of eye shifts between the original panel and the drawing panel during the drawing task was calculated for each trial. In general, the vertical border between the original panel and the drawing panel was used to determine if the gaze crossed from the one panel to the other. On occasions for trials in which the start zones were close to the border, drift of the eye-tracking calibration meant that the gaze position would be incorrectly labeled as being in one panel, when in fact it was in the other, as was determined by visual inspection of the sequence of fixations during a trial. The border was manually adjusted in these instances, and either one eye's data or the average eye data were used, as appropriate.

After categorizing gaze as being on either the original (right) or on the drawing (left), the gaze ratio (G) was calculated for each trial as the total gaze duration during the trial on the original panel versus the total gaze duration on the drawing panel. No attempt was made to analyze individual fixation positions or durations, or to separate fixations, saccadic and smooth pursuit movements.

During the drawing task, participants drew the outline contour of the images nearly continuously. However, from time to time they stopped drawing and did not move the drawing stylus. In order to investigate participants' eye–hand coordination while they were actively drawing, we defined periods of drawing when their stylus speed was higher than 1.5 mm/s or 0.14°/s. The drawing ratio (D) was also computed, as the total of the gaze durations on the original panel, within all active drawing periods within each trial, versus the duration to gaze duration on the drawing panel during the same periods of active drawing. Differences between G and D are due to period of ‘blind’ drawing, where the eyes are on the original, while drawing takes place without central vision (Tchalenko and Miall, 2009).

Average drawing speeds (mm/s) were also computed for each trial. The total length of drawing was calculated for the entire duration from the beginning of active drawing till the end of active drawing. If participants lifted the drawing stylus and continued drawing after a short interval, the interval was deducted from the entire duration.

Procrustes analysis (Kendall, 1989) was used to determine the accuracy of the drawings, conducted using Matlab (version 7.8). Procrustes determines a linear transformation (with translation, orthogonal rotation and scaling) to best conform one data set to another, and reports a goodness of fit (or dissimilarity score) based on the sum of the squared distances between the fitted data points. To conduct the Procrustes analysis, each original image outline was carefully digitized by hand, and the segment of original outline that best represented the section drawn by the participant from the start position to the end of the trial was estimated by eye. The drawn line and the corresponding segment of the original outline were then spatially resampled to have 100 data points each. The Procrustes function returned the goodness of fit between these two sets of data points. Procrustes also reports the rotation, translation and scaling used in the transformation; mirror reversal of the data during the transform was disallowed.

### Scanning protocol

Functional MR imaging was carried out using a 3 T Philips Achieva with eight channel parallel head coil and a sense factor of two. 52 contiguous axial slices were obtained in an ascending order to cover the whole brain, using a gradient-echo echo planar imaging (EPI) sequence (80 × 80 acquisition matrix, field of view of 240 × 240 × 156 mm, 3 × 3 × 3 mm voxel size, and TE = 35 ms, TR = 3000 ms, flip angle = 85°). 357 volumes plus two dummy volumes were acquired for each run which lasted 18 min. A high resolution T1-weighted structural image (1 × 1 × 1 mm, sagittal orientation) was obtained between the first and second runs with a 5 minute MPRAGE sequence.

### fMRI data analysis

fMRI data were analyzed in FEAT v5.98 using FMRIB Software Library package (FSL 4.1.8, FMRIB, Oxford University; Smith et al., 2004, Woolrich et al., 2009; see the FSL website for details: http://www.fmrib.ox.ac.uk/fsl). The Brain Extraction Tool (v2.1) was run on the structural images to extract the brain from the image of the skull and adjoining tissue before running FEAT. Each voxel's time series were corrected to the middle point of the TR, and motion correction was applied to remove the effect of participant's head motion using MCFLIRT (FMRIB's Linear Image Registration Tool). Each EPI image was registered to the middle image of the acquisition set applying a 6 DoF rigid-body spatial transformation. One of the two functional runs for one participant was removed from the analysis because of excessive head motion.

A brain mask from the first volume in the fMRI data was made using BET brain extraction to remove invalid voxels in the fMRI data. Each volume of fMRI data was smoothed with a spatial low-pass filter using a 5 mm full width half maximum (FWHM) Gaussian kernel to lower noise without diminishing valid activation. Low frequency noise was also removed using a Gaussian-weighted high-pass temporal filter with a 180 sec cut-off.

A GLM model was constructed using FILM (FMRIB's Improved Linear Modeling) with prewhitening and with the translation and rotation motion correction parameters as covariates of no interest. These covariates were orthogonalized to one another and all the main experimental conditions. Eight conditions were modeled, and the ‘Rest’ condition was left unmodeled as baseline. The 8 explanatory variables were: face-line (FL), face-silhouette (FS), face-defined (FD), face-undefined (FU), abstract-line (AL), abstract-silhouette (AS), abstract-defined (AD), abstract-undefined (AU). The first 3 s of each trial, during which participants moved the drawing stylus into the start zone, was also separately modeled. All the 9 modeled conditions and their temporal derivatives were convolved with a hemodynamic response function from a gamma function (phase of 0 s, SD of 3 s, mean lag of 6 s).

Registration of each run to a standard space was conducted through a two-stage process. Initially, the motion corrected functional images were registered to the MPRAGE structural using a 6 degrees of freedom (DoF) affine transformation, and the structural image in turn was registered to the MNI standard brain image (MNI152 T1 2 mm) using a 12 DoF affine transformation.

At the first-level of the analysis, each contrast (e.g. faces versus abstracts) was calculated for each individual run of each participant. Contrasts were performed for faces-abstract, for abstract-faces, for undefined–defined trials, and for a linear trend across formats (line, silhouette, defined and undefined, with a contrast vector of − 3, − 1, + 1, + 3). At the second-level of the analysis, the results from the first level analyses for each participant were combined to create a participant average for each contrast (e.g. faces versus abstracts), calculated using a fixed-effects analysis. At the third-level, the participant mean contrast was combined across the group using FLAME (FMRIB's Local Analysis of Mixed Effects) stage 1 and stage 2 (Beckmann et al., 2003, Woolrich, 2008, Woolrich et al., 2004). Z (Gaussianised T/F) statistic images were generated and significant activity identified using clusters determined by Z > 2.3 and a (corrected) cluster defining threshold of p = 0.05 (Worsley, 2001).

The clusters identified from the group analysis were used to create masks for a region of interest analysis. Using the Featquery tool (FMRIB, Oxford; see the FSL website for details: http://www.fmrib.ox.ac.uk/fsl/feat5/featquery.html), the masks were applied to extract mean % signal change of the conditions of interest (FL, FS, FD, FU, AL, AS, AD and AU) for each run across all participants. These mean % signal changes were calculated relative to the mean activation of the ‘Rest’ condition (baseline) in the mask area.

The anatomical locations of clusters were identified using comparisons between a neuroanatomical atlas (Duvernoy, 1999), Harvard–Oxford atlases (Desikan et al., 2006) and the MNI structural atlas (Mazziotta et al., 2001). Neighboring coordinates of the local maxima were used to identify the labels for Brodmann areas in Talairach Daemon Labels included in FSLView 3.1.8 (FMRIB, Oxford; see the FSL website for details: http://www.fmrib.ox.ac.uk/fsl/fslview; Lancaster et al., 2000).

## Results

### Drawing behavior—eye movements

**Fig. 4:**
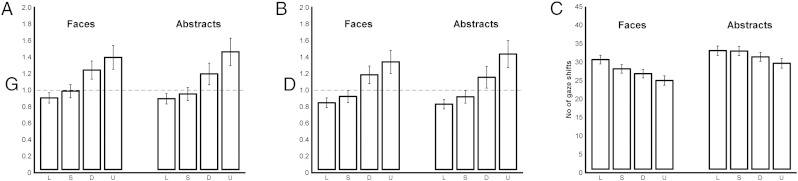
Analysis of gaze behavior. A: G ratio of gaze duration on the original panel to gaze duration on the drawing panel. B: D ratio of gaze duration on the original panel to gaze duration on the drawing panel during the active drawing (drawing speed > 1.5 mm/s). C: Number of eye shifts between the original and the drawing panel. All bars represent group means (n = 14), and error bars represent SEM.

To quantify the change in eye movements across the conditions, we first calculated the gaze ratio (G) of gaze duration on the original to gaze duration on the drawing panel (Fig. 4A). If G is greater than 1, participants look at the original panel longer than the drawing, and we hypothesized that this would correlate with the difficulty of the decision process. Thus, a 2-way repeated measures ANOVA with 2 (type: faces, abstracts) × 4 (format: line, silhouette, defined, undefined) levels was performed. Since Mauchly's test indicated that the assumption of sphericity had been violated, corrected values of degrees of freedom were calculated using Greenhouse-Geisser estimates of sphericity, in this and subsequent ANOVA tests.

There was no significant difference between faces and abstracts (F_(1, 13)_ = 0.01, p = .920, η_p_^2^ = .001). However, there was a significant main effect of format (F_(1.43, 18.62)_ = 3.77, p < .001, η_p_^2^ = .66). Post-hoc tests confirmed that the G ratio in the line task was significantly lower than in both defined (p = .001) and undefined (p < .001), but not different from silhouette (p = .086). G-ratio for silhouette was significantly lower than for both defined (p = .009) and undefined (p = .001), and for defined it was significantly lower than for undefined (p = .012). There was no significant interaction between type and format (F_(3, 39)_ = 1.16, p = .338, η_p_^2^ = .09). These results confirm the hypothesis that the time spent on the original varied with the stimulus formats (Fig. 4A), and suggest the difficulty in decisions about the drawing task rose from line to silhouette to defined to undefined.

The drawing ratio (D) of gaze duration on the original panel to gaze duration on the drawing panel during periods of active drawing (drawing speed > 1.5 mm/s or 0.14 °/s) was also calculated (Fig. 4B). Just as for the G-ratio, if D was greater than 1, participants looked at the original panel longer than the drawing panel, while actively drawing. We hypothesized from other work (Tchalenko et al., 2014) that this drawing ratio would be similar to G, as drawing rates would remain constant despite change in time viewing the original, and increasing amounts of “blind drawing”, i.e. active drawing while looking at the original, would take place as drawing decisions increased. A 2-way rmANOVA with 2 (type: faces, abstracts) × 4 (format: line, silhouette, defined, undefined) levels was conducted on the D-ratio.

There was no significant difference between faces and abstracts (F_(1, 13)_ = 0.03, p = .856, η_p_^2^ = .003). However, there was a significant main effect of format (F_(1.50, 19.46)_ = 23.84, p < .001, η_p_^2^ = .65, Greenhouse-Geisser corrected). Post-hoc tests illustrated that the ratio was significantly lower in line than defined (p = .001) and undefined (p < .001), but not different from silhouette (p = .099). Silhouette was significantly lower than defined (p = .013) and undefined (p = .002), and defined was significantly lower from undefined (p = .016). There was no significant interaction between type and format (F_(1.96, 25.50)_ = 1.25, p = .305, η_p_^2^ = .09, Greenhouse-Geisser corrected). Thus, these results also confirm our hypothesis—even during active drawing periods, greater time was spent with gaze on the original panel in those tasks assumed to require greater decisions, in ascending order of line, silhouette, defined and undefined (Fig. 4B).

We next tested the number of eye shifts between the original panel and the drawing panel (Fig. 4C); as above, from our previous work we predict fewer shifts for the more difficult drawing decisions, as longer time is spent in each dwell period (Tchalenko et al., 2014). Significantly less shifts were made for faces compared to abstracts (F_(1, 13)_ = 84.56, p < .001, η_p_^2^ = .87). There was also a significant main effect of format (F_(3, 39)_ = 84.99, p < .001, η_p_^2^ = .87) and a significant interaction between type and format (F_(3, 39)_ = 6.53, p = .001, η_p_^2^ = .33). A simple main effects analysis showed that the number of gaze shifts significantly decreased in the order of format (line > silhouette > defined > undefined) for both faces (p ≤ .027) and abstracts (p ≤ .044), except for between line and silhouette formats for abstract stimuli (p = 1.000). Thus consistent with the G- and D-ratios, fewer gaze shifts were seen in the conditions defined and undefined, assumed to be the more difficult ones, as gaze dwelt longer on the original panel than on the drawing panel.

In summary, both faces and abstracts induced a near-identical pattern of G- and D-ratios; both ratios were higher for defined and undefined formats than for line and silhouette formats, and the ratios for undefined were higher than for defined. Participants made almost the same number of gaze shifts for both faces and abstracts, and the number of gaze shifts decreased in the order of line, silhouette, defined and undefined, except for between line and silhouette of abstracts.

### Drawing behavior—hand movements

We quantified the match between the drawn line and the original for every trial, and monitored the average speed of the drawing during all active drawing periods. We had no a priori hypotheses about how accuracy would be affected by stimulus format—difficult drawing decisions might be compensated by changes in gaze behavior, as described above, such that the final accuracy was equivalent despite increased difficulty. From the Procrustes analysis of drawn line shape, values of dissimilarity, rotation and scale of the transformation from the drawn line to the original contour were computed. Two-way rmANOVAs were then conducted on these parameters. We did not analyze the translational components because the start position of each drawn line was constrained by the fixed start circle, and thus translation was constrained.

**Fig. 5:**
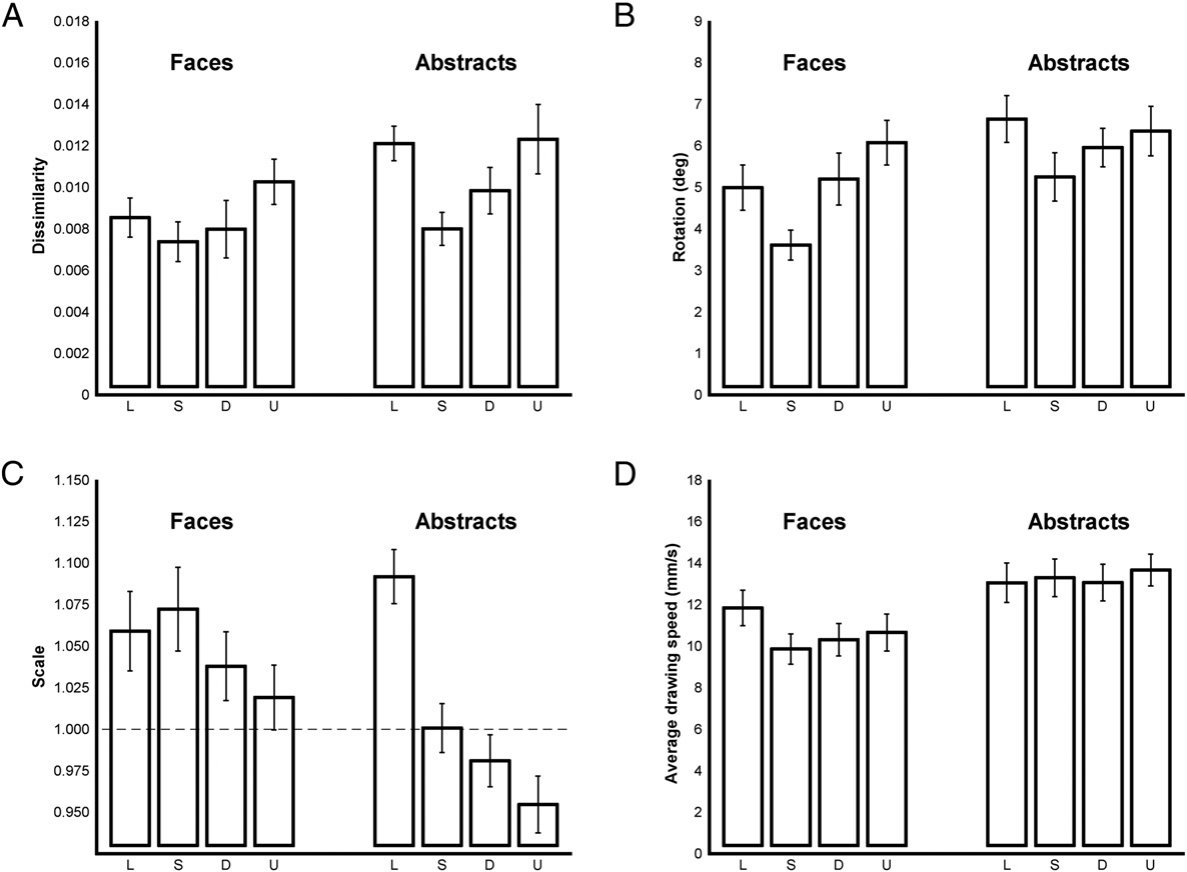
Analysis of drawing behavior. A–C: output of the Procrustes analysis of drawn line shape. A: Values of dissimilarity measure, in which high scores indicate poor shape matching to the original. B: Rotation component, in degrees—a low score indicate less rotation. C: Scale component. If the scale is greater than 1 (dashed line), the drawn outline is larger than the original outline, whereas a scale lower than 1 indicates a smaller drawing. D: Average drawing speed (mm/s) during the drawing task, calculated across all active drawing periods. All data represent group means (n = 14), and error bars represent SEM.

The value of dissimilarity was significantly smaller for faces compared to abstracts (F_(1, 13)_ = 7.07, p = .020, η_p_^2^ = .35, Fig. 5A). High dissimilarity scores indicate less accurate overall fit of the shape between the original and the drawn line. Thus overall there was a better match of shape in the faces condition than in the abstract condition. There was also a significant main effect of format (F_(3, 39)_ = 6.56, p = .001, η_p_^2^ = .34), but there was no significant interaction between type and format (F_(3, 39)_ = 1.00, p = .403, η_p_^2^ = .07). Bonferroni-adjusted post-hoc t-tests illustrated that the value of dissimilarity measure for silhouette was significantly smaller than line (p = .027, Cohen's d = 0.59) and undefined (p < .007, Cohen's d = 0.79). The dissimilarity of defined was significantly smaller than undefined (p = .013 Cohen's d = 0.63).

The rotation error was significantly greater for abstracts compared to faces (F_(1, 13)_ = 10.05, p = .007, η_p_^2^ = .44, Fig. 5B). There was also a significant main effect of format (F_(3, 39)_ = 4.43, p = .009, η_p_^2^ = .25), but no significant interaction between type and format (F_(3, 39)_ = 1.02, p = .394, η_p_^2^ = .07). Bonferroni-adjusted post-hoc tests illustrated that the rotation error for silhouette was significantly smaller than defined (p = .015) and undefined (p < .012).

The scale component was greater than 1 if the drawn outline was bigger than the original outline (Fig. 5C). The results showed that faces were drawn at a larger scale than abstracts (F_(1, 13)_ = 7.13, p = .019, η_p_^2^ = .35). There was also a significant effect of format (F_(3, 39)_ = 44.70, p < .001, η_p_^2^ = .78) and a significant interaction between type and format (F_(3, 39)_ = 20.13, p < .001, η_p_^2^ = .61). A simple main effects analysis illustrated that silhouette, defined and undefined faces were drawn at a significantly larger scale than silhouette, defined and undefined abstracts (t(13) ≥ 2.94, p ≤ .011, Bonferroni-adjusted). However, the scale of abstract line drawings was significantly larger than that of line faces (p = .036), and greater than all other conditions.

The average drawing speed (mm/s) was significantly slower for faces compared to abstracts (F_(1, 13)_ = 67.79, p < .001, η_p_^2^ = .84; Fig. 5D). There was a significant main effect of format (F_(1.73, 22.50)_ = 11.08, p < .001, η_p_^2^ = .46, Greenhouse-Geisser corrected) and also a significant interaction between type and format (F_(3, 39)_ = 17.08, p < .001, η_p_^2^ = .57). A simple main effects analysis showed that the average drawing speeds of each level of format for faces were significantly slower than for abstracts (t(13) ≥ 6.65, p ≤ .001). Post-hoc tests for the faces showed that drawing speed was higher in the line condition than in all other conditions (p < .015, Bonferroni-adjusted), and that undefined was faster than silhouette (p = .038). For abstracts, the only difference was that undefined was faster than defined (p = .036).

In summary, there was no simple relationship between parameters describing the drawn lines and the assumed decision difficulty. The similarity of the drawn line shape to the original was greater for faces than for abstracts, indicating better shape matching. The rotation component varied across the four formats, but the mean was within about 3–7 degrees. The slightly greater mean rotation for the abstract stimuli may reflect that they have no obvious natural orientation (Fig. 1). In general the scale of the face drawing was greater than one, and also greater than for the abstracts, while the scale for the abstract line was bigger than for all other conditions. Note however that all mean scale values are within 5–10% of unity. Finally, the average drawing speed was slower for faces than for abstracts for all formats, and showed differences between the different face formats that were not evident between the abstract formats. For the faces, drawing speed was lowest for the silhouette condition, and highest for the line condition.

Finally, in relation to the interpretation of the functional activation data, the differences in hand actions across conditions (movement scale and drawing speed) were relatively small, under 10% in all cases except for a 15% difference in scale of drawing for the abstract line versus abstract undefined conditions. Even in this extreme case, there was no difference in drawing speed, a factor that might influence motor execution of the hand action.

### Functional activations

We hypothesized that the decisions about how to draw each image would be affected by the stimulus type and also by the stimulus format. We therefore tested for a main effect of stimulus type (face vs. abstract) and secondarily for a linear trend across the four stimulus formats (in order of assumed difficulty, line, silhouette, defined and undefined). We further hypothesized that there would be an interaction between these two factors, such that decisions about difficult face drawing conditions might differentially engage cognitive processes compared to easier abstract drawing conditions. We therefore conducted a further ROI analysis based on the results of the primary analyses. Finally we performed a contrast of the undefined versus defined format conditions, to test for greater activity in the former, more challenging condition.

#### Faces versus abstract objects

**Fig. 6:**
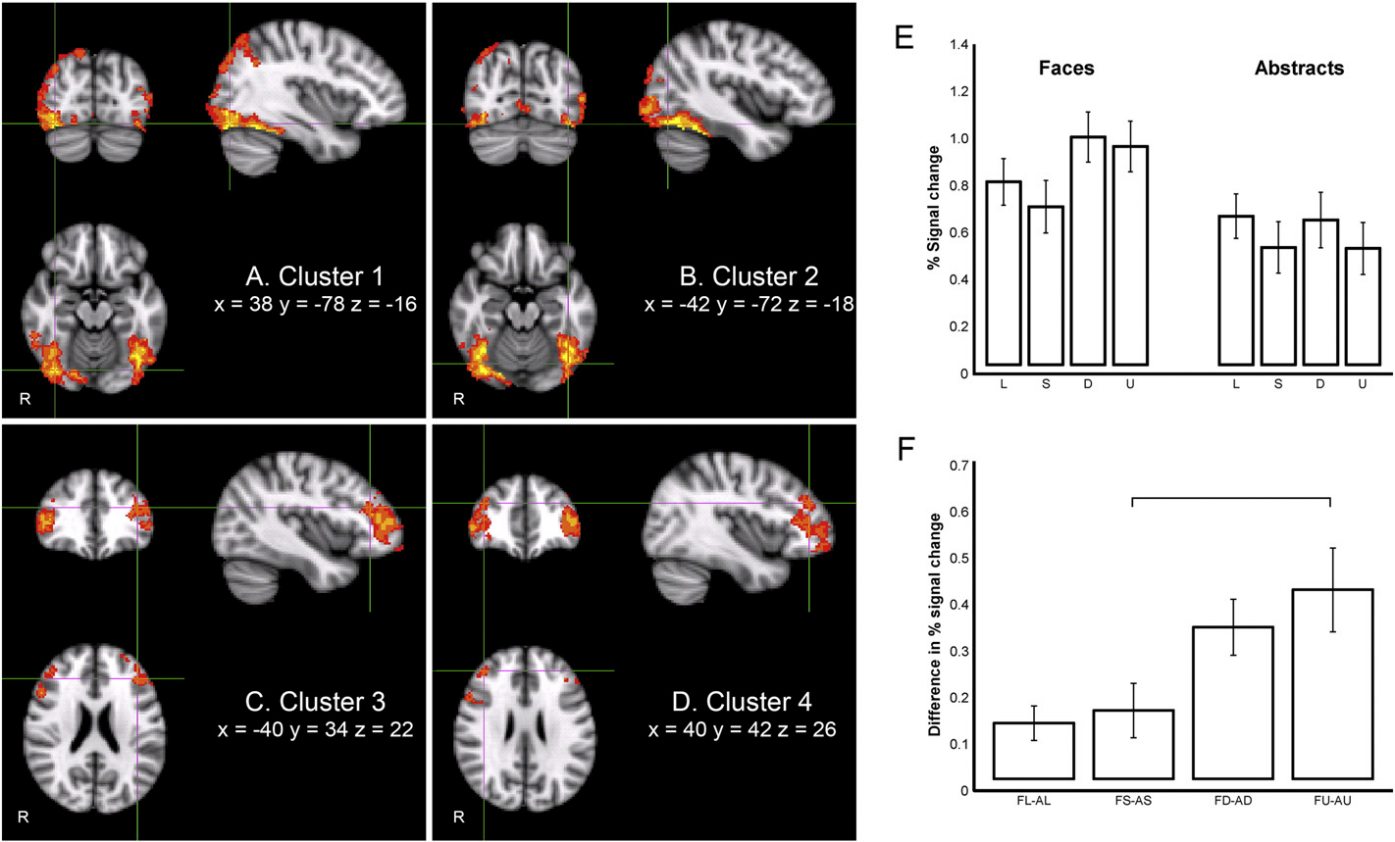
Four clusters identified by their significantly greater activation in faces than in abstract conditions, and results of region of interest analysis. The anatomical locus of each cluster is shown in Table 1. The cross-hairs indicate local maxima of FFA (A and B) and MFG (C and D). E: Mean % signal changes of line, silhouette, defined, undefined of faces and abstracts are extracted from the cluster shown in Figs. 6A–B, constrained by a mask comprising bilateral FFA, LO and ITG ( 1 ± SEM, n = 14). F: Magnitude of differences in this region between faces and abstracts at each level of format (FL-AL, FS-AS, FD-AD, FU-AU). Silhouette and undefined are significantly different and the differences between line and defined and line and undefined approach significance. All data represent group means (n = 14), and error bars represent SEM.

We first performed a main contrast of face trials (FL, FS, FD, FU) versus abstract trials (AL, AS, AD, AU). Four clusters showing stronger activation for drawing faces relative to abstract objects were identified from the group analysis (Figs. 6A–D). Two of these clusters in the occipital and temporal lobes were composed of bilateral fusiform face area (FFA, both occipital and temporal areas), bilateral lateral occipital cortex (LO, both inferior and superior areas), bilateral inferior temporal gyrus (ITG), bilateral lingual gyrus, right superior parietal lobe (SPL) and right precuneus (Table 1, Fig. 6, clusters A, B). The remaining two clusters lay in the frontal lobe and were composed of bilateral frontal pole (FP), bilateral middle frontal gyrus (MFG) and bilateral inferior frontal gyrus (IFG) (Table 1, Fig. 6, clusters C, D).

**Table 1:** Four clusters (A–D) identified from the contrast between faces and abstracts, based on Z > 2.3 and a corrected cluster defining threshold of p = .05.

Cluster ID	Cluster vol (cc)	Cluster p	Z	Coordinates (mm)			BA	Anatomical locus
				x	y	z		
A	108.8	< .00001	4.75	38	− 78	− 16	19	FFA (occipital) R
			4.38	38	− 58	− 20	37	FFA (temporal) R
			4.69	40	− 80	− 8	18	Lateral occipital (inferior) R
			3.48	44	− 82	20	19	Lateral occipital (superior) R
			3.75	60	− 44	− 14	20	Inferior temporal gyrus R
			3.91	32	− 64	42	19	Superior parietal lobe R
			3.22	14	− 78	52	7	Precuneus R
			3.43	2	− 66	6	18	Lingual gyrus R
			2.89	− 6	− 72	− 2	18	Lingual gyrus L
B	105.3	< .00001	4.74	− 42	− 72	− 18	19	FFA (occipital) L
			4.69	− 46	− 58	− 22	37	FFA (temporal) L
			3.82	− 44	− 88	− 6	18	Lateral occipital (inferior) L
			3.29	− 48	− 80	20	19	Lateral occipital (superior) L
			3.78	− 56	− 58	− 24	37	Inferior temporal gyrus L
C	43.2	< .00001	4.18	− 30	62	− 6	10	Frontal pole L
			3.31	− 40	34	22	9	Middle frontal gyrus L
			3.13	− 50	36	12	46	Inferior frontal gyrus L
D	40.7	< .00001	3.32	32	60	− 10	10	Frontal pole R
			3.22	40	42	26	9	Middle frontal gyrus R
			3.86	50	36	4	46	Inferior frontal gyrus R
E	618.4	< .00001	4.57	30	− 34	− 54	3	S1 R
			3.59	22	− 18	− 62	4	M1 R
			4.56	2	− 86	16	18	Cuneus R
			4.36	18	− 48	48	7	Precuneus R
			4.18	− 28	− 32	52	3	S1 L
			4.44	− 26	− 26	58	4	M1 L
			4.00	− 8	− 88	24	18	Cuneus L
			3.88	− 8	− 48	52	7	Precuneus L

Local maxima and MNI-space coordinates of anatomical loci of the clusters are identified. Bilateral local maxima (E) at a raised threshold of Z > 3.0 are reported from the contrast between abstracts and faces. Brodmann areas (BA) were identified with Talairach Daemon Labels included in FSLView 3.1.8.

For the reversed contrast (abstracts versus faces), a number of small activations were found (Z > 2.3) in several cerebral areas across both hemispheres. Table 1E reports only the bilateral local maxima that survived at a raised threshold of Z > 3.0. We do not focus on this reverse contrast for two reasons. First, our hypothesis was driven by the idea that enhanced knowledge of the face category will provide addition input to the perceptual decision processes, thus arguing for a faces-abstract contrast. Second, the contrast revealed an extensive activation in a single cluster spanning much of the sensory–motor areas, involving both hemispheres, but also extending into the cuneous and precuneus (Table 1E). There was no a priori reason to expect such extensive activity, and we failed to see a clear pattern within the areas activated that leads to a functional interpretation.

To further explore the differences in activation levels in the main clusters (Table 1A–D), region of interest analyses were conducted using these four functionally identified clusters constrained by their overlap with anatomically derived masks, limiting the ROI to ventral temporo–occiptial regions. One mask therefore included bilateral FFA, inferior LO and ITG (Fig. 6), but excluded the superior LO, parietal and lingual activations. Fig. 6E shows the mean activation level for the ventral region including bilateral FFA, inferior LO and ITG across the 8 conditions.

A 2 (type: faces, abstracts) × 4 (format: line, silhouette, defined, undefined) repeated measures ANOVA was carried out on mean % signal changes extracted from this first region (FFA, LO and ITG; Fig. 6E). As expected from the identification of these regions as more active in face than abstract condition, face trials resulted in significantly stronger activation than abstracts (F_(1, 13)_ = 71.17, p < .001, η_p_^2^ = .85). A simple main effects analysis showed that faces led to significant activation compared to abstracts under all four different formats (t(13) ≥ 2.94, p ≤ .012; Fig. 6E). There was also a significant main effect of format (F_(3, 39)_ = 10.53, p < .001, η_p_^2^ = .45), with noticeably higher activation in the defined and undefined formats for faces (FD and FU) than for other conditions. In addition, the interaction between type and format was significant (F_(1.82, 23.67)_ = 4.70, p = .022, η_p_^2^ = .27, Greenhouse-Geisser corrected). The effect sizes reported here for stimulus category should be interpreted with caution, since the voxels for this analysis were selected using a linear contrast of stimulus category. Results for stimulus format, on the other hand, are unaffected by this potential bias, because stimulus format was completely orthogonal to the stimulus category variable. One way ANOVA conducted on the differences in mean percentage signal changes between faces and abstracts at each level of format (FL-AL, FS-AS, FD-AD, FU-AU) illustrated that there was a significant linear trend in the magnitudes of difference (F_(1, 13)_ = 18.55, p = .001, η_p_^2^ = .59; Fig. 6F). In other words, the activation difference between faces and abstract stimuli increased, in order of assumed difficulty, from line, silhouette, defined to undefined formats (Fig. 6F). Post-hoc tests using a Bonferroni adjustment showed that the magnitude of difference between silhouette (FS-AS) and undefined (FU-AU) was significantly different (p = .013). The differences between line (FL-AL) and defined (FD-AD), p = .068, and between line (FL-AL) and undefined (FU-AU), p = .057, approached significance.

The additional region of interest analyses within the network of areas more active in the faces condition, anatomically constrained by its overlap with the right SPL and right precuneus, only confirmed that faces led to stronger activation relative to abstracts in those regions (F_(1, 13)_ = 26.84, p < .001, η_p_^2^ = .67), bilateral lingual gyrus (F_(1, 13)_ = 12.34, p = .004, η_p_^2^ = .49), bilateral FP, MFG and IFG (F_(1, 13)_ = 40.30, p < .001, η_p_^2^ = .76), but did not expose significant differences across formats, or interactions between type and format.

#### The effect of presentation format

**Fig. 7:**
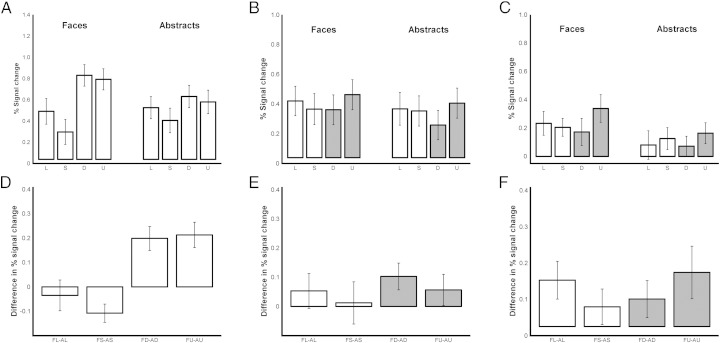
Results of supplementary region of interest analyses. A–C: Mean % signal changes of line, silhouette, defined, undefined of faces and abstracts extracted from clusters identified from the linear trend contrast across the stimulus formats (A) and from the contrast of undefined versus defined presentation formats (B: right hemisphere, C: left hemisphere). In this contrast, regions were identified with significant activity differences between the two grey bars only. D–F: Mean differences between faces and abstracts in each format (i.e. FL-AL, FS-AS, FD-AD, FU-AU), for the corresponding data in the top row (A–C). All data represent group means (n = 14), and error bars represent SEM.

One additional network of interest was identified from the group analysis after conducting a main contrast of a linear trend across the four formats (with contrast weights of − 3, − 1, + 1 and + 3 for line, silhouette, defined and undefined, respectively). It encompassed bilateral FFA (both occipital and temporal areas), bilateral occipital pole (OP), bilateral lingual gyrus and bilateral LO which was marginally extended from OP, very similar to the cluster shown in Fig. 6. The mean activity within this network, across the 8 conditions (Fig. 7A), had a very similar profile as seen in Fig. 6E, although the difference in activity between the four faces formats was greater (Fig. 7A). Hence, while the aim of this contrast was to identify areas changing activity in a linear fashion, in fact the only regions that were found showed greater activation in the defined and undefined conditions, but actually also showed greater activation in the line format than in the silhouette format.

A 2 (type: faces, abstracts) × 4 (format: line, silhouette, defined, undefined) rmANOVA was carried out on the mean BOLD signal change in these regions. Faces led to significantly stronger activation than abstracts (F_(1, 13)_ = 8.43, p = .012, η_p_^2^ = .40). There was a significant main effect of format (F_(3, 39)_ = 60.57, p < .001, η_p_^2^ = .82). There was also a significant interaction between type and format (F_(3, 39)_ = 9.40, p < .001, η_p_^2^ = .42). A simple main effects analysis illustrated that for faces, both defined and undefined respectively led to greater activations compared to both line and silhouette (p ≤ .001), and line was greater than silhouette (p = .0.12). For abstracts, both defined and undefined resulted in greater activations compared to silhouette (p ≤ .009). However, unlike the results shown in Fig. 6F, the differences between the faces and abstracts conditions were driven mainly by a stronger signal in the two photographic formats (defined and undefined) compared to the other formats (line and silhouette). In other words, the mean signal in this region was almost identical for the face and abstract stimuli in line format, while the silhouette format of abstracts led to somewhat greater activation than that of faces. However, the mean signal was significantly greater for faces both in the defined and undefined formats than for abstracts (FL = AL, FS < AS, FD > AD, FU > AU).

#### Defined vs. undefined formats

Finally, in order to test for any additional differences in activation when we had experimentally manipulated the contrast of the two photographic image formats, to make the line decisions more ambiguous, we performed a contrast between the undefined versus defined conditions. Two clusters were identified as significant from this group analysis. One cluster on the right hemisphere included IFG, temporal pole (TP), MFG and insula (Fig. 7B) while the other cluster on the left hemisphere included FP and MFG (Fig. 7C). Both showed stronger activation in the undefined condition.

A 2 (type: faces, abstracts) × 4 (format: line, silhouette, defined, undefined) rmANOVA was carried out on the mean activity for the regions in the right hemisphere (Fig. 7B). There was no significant difference between faces and abstracts (F_(1, 13)_ = 2.88, p = .113, η_p_^2^ = .18), and there was no significant interaction between faces and abstracts (F_(3, 39)_ = .41, p < .727, η_p_^2^ = .03). However, the significant main effect of formats (F_(3, 39)_ = 6.34, p = .001, η_p_^2^ = .33) revealed the difference between defined and undefined (p = .001), for which this cluster had been identified. In both faces and abstract stimuli, there was a similar 0.1% increase in BOLD activation for the undefined format (FU > FD, AU > AD), a difference of about a quarter of the mean signal seen in the other conditions.

The same 2 (type: faces, abstracts) × 4 (format: line, silhouette, defined, undefined) rmANOVA was also carried out on the mean BOLD from the regions on the left hemisphere (Fig. 7C). Here, faces led to significantly greater activation than abstracts (F_(1, 13)_ = 12.32, p = .004, η_p_^2^ = .49). The significant main effect of formats (F_(3, 39)_ = 4.32, p = .010, η_p_^2^ = .25) showed the expected difference between defined and undefined (p = .007). However, there was no significant interaction between faces and abstracts (F_(3, 39)_ = .75, p < .527, η_p_^2^ = .06). In this region the difference between defined and undefined formats represented about 80% of the mean signal in the other conditions (FU > FD, AU > AD).

To explore potential relationships between behavioral measures and the BOLD contrast between drawing conditions, we performed post-hoc regression between the difference in drawing scale and accuracy (as measured by the Procrustes analysis) and the mean activation differences between faces and abstract conditions, for the four identified ROIs, across the 14 participants in the group. There were no significant relationships for any of the ROIs (p > 0.102). We did however, find a significant and positive relationship for the frontal ROI (r = 0.3, p = 0.018 DOF = 54, for ROI including the cluster shown in Figs. 6C,D) and for the lingual ROI (r = 0.36 p = 0.006 DOF = 54). There was a marginally significant relationship for the parietal ROI (r = 0.27 p = 0.045 DOF = 54); the regression was not significant for the ventral occipital ROI including the cluster shown in Figs. 6A,B. These relationships suggest a negative relationship between speed and BOLD activation, as the speed was higher in abstract than faces, while the clusters reported were based on greater activation in faces compared to abstract. We are cautious in our interpretation, as our experimental design did not aim to induce great variation within the behavioral measures, although there was in fact a difference in speed between the different drawing trials. Our group size is also small for such within-group regression analyses. However, it is clear that this negative relationship does not explain the interaction effects reported in Fig. 6F: the relationship with the individual's speed was not significant for this region, and in fact the differences in group mean speed between conditions (Fig. 5D) does not conform to the BOLD activations reported.

## Discussion

This experiment aimed to isolate the neural systems involved in making decisions about how to draw observed pictures. We recorded eye and pen movements during whole brain functional imaging, and found that presentation of images of faces or abstract objects to be drawn in four different picture formats led to significant differences in eye movements and drawing metrics. We worked from the assumption based on our previous behavioral analyses (Tchalenko et al., 2014) that the difficulty in deciding on a line to be drawn would increase across line, silhouette, defined and undefined formats. As predicted from this, both the gaze ratio and the drawing ratio increased in order across the four presentation formats. The number of eye shifts between the original and the drawing panels of the display showed the opposite pattern. Thus when the discrimination of the prescribed feature of the stimulus image was made most difficult (i.e. for the undefined photographs with their deliberately reduced contrast), participants spent longer—up to 40% more—viewing the original and shifted their eyes between the original and the drawing panel less frequently (Fig. 4A). This was the case even during periods of active drawing (as captured by the D-ratio, Fig. 4B, the ratio of active drawing time spent looking at the original image instead of the drawing).

In contrast to these obvious and clearly ordered changes in ocular behavior, there were less orderly changes in the drawing behavior (Fig. 5). There was an overall trend for our quantitative measures of inaccuracy in drawing (the rising dissimilarity and rotation scores) to follow the same order of changes seen in ocular metrics especially across silhouette, defined and undefined formats. The dissimilarity scores reported by Procrustes analysis of the drawn lines appear small (below 0.015 or 1.5%, implying very high accuracy), but these scores closely match those seen in other work (Tchalenko et al., in preparation). For example, in that study, dissimilarity scores reached about 0.007 (0.7%) when participants copied complex lines with a separation between original and copied lines of 15 degrees, similar to the 16 degree separation between original and picture panels in the present study. They found dissimilarities of 0.002 (0.2%) for direct tracing over a complex line: this is about 4 times smaller (more accurate) than seen here for the most accurate drawing under silhouette conditions. This value may represent a fundamental accuracy limit in line-copying, governed by visuo–motor control of the pencil. We would expect and did find slightly larger errors under the conditions used here, when participants drew within the constrained environment of the scanner, with indirect visual feedback of the pen motion, and with the line drawing recorded with a relatively low resolution touch panel. However, while these scores are low, the key point is that the relative accuracy between the presentation conditions changed by as much as 50% (Fig. 5A).

Note also that the similar trend in the scale measure (Fig. 5C) for face and abstract conditions, across three formats, silhouette, defined and undefined, actually implies opposite shifts in size—for faces, the scale measure approaches unity, whereas for abstracts, the scale gradually reduces below unity. Thus changes in scale may be independent from similarity and rotation, in terms of an overall accuracy of reproduction. Intuitively, the (dis)similarity measure may be the most important of these metrics. A drawing would be recognizable even if drawn at the wrong scale and orientation, whereas the reverse is not necessarily true. On this basis, considering (dis)similarity as the primary measure of drawing accuracy, there were more accurate drawings completed for silhouette than for the defined formats, and more accuracy in the defined than the undefined stimuli.

The high dissimilarity scores for the lines format were unexpected. It is not clear why this condition leads to less accuracy, higher rotations and—for the abstract line format—a large scaling error. We speculate that in the viewing conditions in the scanner, with indirect cursor feedback of the drawn line, it is easier to correctly locate and scale ones rendition of the other, more complete formats. Further work would be needed to understand this aspect of the drawing performance.

Relating these drawing accuracy measures to the number of gaze shifts (Fig. 4C), we suggest that the gaze shift rate is more closely related to task difficulty (i.e. in the decisions about the original stimulus image) than to the accuracy of performing the drawn line. This last point is supported by the dissociation between the metrics of line drawing and drawing in silhouette, defined and undefined formats. The line presentation format should be the easiest decision task, as there is no ambiguity about what line is to be drawn, and indeed the low G-ratio and the higher number of gaze shifts reflect less time, and shorter dwell time, spent with gaze on the original in this condition than in any other. In contrast, the line format appears to be the exception to the linear trend seen in each measure of drawing performance (Fig. 5). In particular, the abstract stimuli presented in line format were drawn with noticeably less accuracy than expected from the gaze behavior. We cannot yet determine why this should be the case; it is not simply a trade-off between speed and accuracy, because the changes in average drawing speed were not congruent with the changes in drawing dissimilarity. In fact, drawing speed was quite slow in all conditions (mean about 12 mm/s) and did not vary systematically across presentation formats, although it was higher in the abstract condition than in the face condition. We suggest therefore that the dissimilarity, scale and rotation metrics reflect planning errors in drawing, while the drawing speed reflects the relatively constant performance demand of executing the planned lines.

The main focus of the experiment was of course to be able to compare functional activation levels across four presentation formats, driven by our a priori assumption that decisions about the line to be drawn would be modulated by the format. Our eye movement metrics recorded simultaneously confirmed that the participants indeed behaved as if they found the formats to increase in difficulty in order of line, silhouette, defined and undefined. Furthermore, the differences in hand drawing actions across the different conditions were relatively small, accounting to changes in scale of below 7%, and difference in speed of the pen motion of less than 3 mm/s (Fig. 5D). Even the statistically significant differences in ocular control that we observed—the increase in the number of gaze shifts between original to drawing, for example—were in the order of 10% of the average. We were therefore largely successful in keeping overt ocular and manual motor execution parameters approximately equal, and we did not see the sensorimotor and oculomotor areas identified in our main comparison of interest between timulus conditions (faces vs. abstract). However, the reverse contrast (abstract vs. faces) did reveal significant bilateral S1 and M1 activation. This could be due to the approximately 10% greater drawing speed observed for the abstract conditions, despite the low overall speeds. However, this activation pattern did not reflect the differences in drawing speed seen between the four formats.

We did, however, find a broad network of occipito–temporal, parietal and prefrontal regions that were differentially activated when the drawing faces task was contrasted with drawing abstract objects. The object images were generated from photographs of folded white towels (Fig. 1). They superficially resembled the shape of a human head, and in line and silhouette formats had close similarity to the faces stimuli. The order of presentation of the trials was carefully counterbalanced across formats and stimulus type, so there is some possibility that in some trials in which objects were presented, the class of stimulus (face versus object) might have been unknown to the participants. However, the category would have been clear in the vast majority of face stimulus trials, cued by the neck and shoulders, or by the hairline in the line format, and the category would have been unambiguous for all stimuli when presented in the photographic formats, even in the undefined cases with reduced contrast. It is noticeable however, that only the photographic formats had internal features of the human faces, in particular eyes and mouth. Images with these canonical features are known to activate the face-sensitive regions in the fusiform and occipital areas of extrastriate visual cortex (Grill-Spector et al., 2004, Kanwisher et al., 1997, Kourtzi and Kanwisher, 2000). This difference in the facial features is likely to be the cause of the increased activation in ventral areas in the photographic faces conditions (Figs. 6E,A). In contrast, parietal and frontal areas were more engaged by the difficult undefined and defined conditions for both stimulus categories, but were not selectively driven by the facial features.

Turning next to the hypothesized interaction between stimulus types and the presentation formats, the tempero–occiptal–parietal and frontal network that was identified only on the basis of its increased activation for faces (Fig. 6), did indeed show this strong interaction. The difference in activity across this region, especially in the ventral areas consisting of bilateral FFA, LO and ITG showed a strong monotonically increasing difference between faces and abstracts (Fig. 6F). This increasing difference parallels the increase in the ocular D- and G-ratios (Fig. 4), that is, the amount of time spent viewing the original image, rather than with gaze on the drawing panel. We suggest that this reflects the time required for the selection of the line to be drawn and to plan how to draw it, under the different formats. As the activation in this region is higher for faces than for abstracts across all four formats, the results support a domain-specific mechanism in these ventral areas. In other words, the pattern of ocular behavior is common across both stimulus categories (faces and abstracts, Fig. 4), and this alone cannot account for the interaction seen in Fig. 6F. But since the FFA has a strong preference for the faces stimuli, the monotonic increase in its activation over that during the abstract stimuli could be explained by the top-down modulation of this region during the more challenging of the face processing conditions.

We speculate therefore that this difference reflects an increasing influence of top-down areas onto the ventral cortex, priming these extrastriate visual areas to extract the visual features that are to be drawn. When the features are poorly defined, as in the undefined stimuli, prior knowledge of the stimulus category—faces—allows the participant to make the difficult judgments about the line to be drawn. In this study, we have been able to identify the influence of this top-down knowledge on the processing of lower areas only in the category of faces; other more specific classes of stimuli might be needed to indentify similar top-down influence in processing, for example, objects or scenes.

We should acknowledge that our design could be improved. Through our choice of folded towels with typically only one obvious edge or fold, the complexity of the photographic face stimuli is higher than that for the abstracts. By default, only the photographic defined and undefined faces had internal features (eyes, mouth etc), and this might lead to higher interest and attention. Thus the increase in complexity between the two photographic face formats and the other two line and silhouette formats is greater than that seen for the abstract stimuli, and might engage higher interest or attention. We do not believe that this can be the major cause of the activation pattern seen in the tempero–occiptal–parietal areas, however, as an attentional account would suggest longer viewing times for the more engaging stimuli. In contrast, our behavioral measures show that gaze ratios were very closely matched between the two stimulus classes, and all of the ocular metrics showed a linear trend across the 4 formats, for both abstract and face stimuli.

The domain-specific effect that we believe is driving these changes in extrastriate cortical activation levels is closely in line with the model of proposed perceptual advantages for trained artists put forward by Seeley and Kozbelt (2008). They suggested that processing of image features within the visual stream (including V1, MT, V4 and TEO) would be primed by categorical knowledge from the DLPFC and from area TE, to allow extraction of visual features consistent with a current perceptual hypothesis, but also by motor working memory or action schemata from motor areas such as rostral SMA and PMC, that would constrain visual processing and direct visual attention consistent with planned motor acts. We did indeed find significant activation of middle and frontal gyrus, bilaterally. We also found activation of the frontal pole, BA10, and this may be consistent with Seeley and Kozbelt (2008)'s hypothesis that high levels of the motor hierarchy constrain visual processing though their role in planned motor acts. This area can be activated by high-level decisions about the value of actions (Ramnani and Miall, 2003).

In summary, we have shown that line drawing of facial features in visual images engages a number of lateral and ventral occipital areas, and the activation in these areas may reflect both their domain-specific sensitivity for faces, and their top-down modulation by other cortical areas—perhaps prefrontal—in agreement with theory about perceptual ability in trained artists. Our study has only tested untrained participants, and while trained artists might be expected to have developed particular experience in making judgments on visual images, normal healthy adults would have significant domain-specific knowledge about faces. The tempero–occipital activation therefore may reflect top-down knowledge that the participants are using to disambiguate the drawings (knowledge that can distort perception at times). Solso (2001) has reported a pilot study comparing one skilled artist with untrained participants, and indeed showed higher frontal activation, and less occipital activity in the artist. Interestingly, Chamberlain et al. (2014) have just reported an anatomical study with trained and untrained artists that suggests increased grey-matter in right medial frontal gyrus correlated with observational drawing ability (cf. Table 1D), while right precuneus correlated with observational drawing training (cf. Table 1E). A full functional study comparing artists with non-artists would be a valuable extension to our work, to further clarify this issue.
